# Molecular detection of *Coxiella burnetii* in blood and hard tick-infested Egyptian camels and the possibility of coinfections

**DOI:** 10.1007/s11250-024-04131-7

**Published:** 2024-10-10

**Authors:** Radwa Ashour, Dalia Hamza, Mona Kadry, Maha A. Sabry

**Affiliations:** Department of Zoonoses, Faculty of Veterinary Medicine, Cairo University, Giza, 12211 Egypt

**Keywords:** *Coxiella burnetii*, Q fever, Coinfection, IS111 gene, *Borrelia* spp., *Babesia microti*

## Abstract

*Coxiella burnetii*, a bacterium that causes Q fever. It can infect mammals and has a global geographical distribution, but data on its occurrence in Egyptian dromedaries and the associated ticks are limited. Therefore, this study aims to detect *C. burnetii* in the blood of infested camels and associated ticks collected from Egypt by using molecular techniques and to examine the possibility of coinfections with *C. burnetii.* A total of 133 blood samples and 1260 hard ticks infesting these camels were collected from Egyptian slaughterhouses. Nested PCR and sequencing were used based on the IS1111 gene for molecular detection of *C. burnetii.* The identification of tick species at the molecular level was performed using the *COX1* gene. *C. burnetii* was detected in *Hyalomma (H) dromedarii*,* H. anatolicum*,* H. marginatum*,* Amblyomma (Am) lipidium*, and *Am. cohaerens* with an overall prevalence rate of 1.3% (16/1260), while in the camel blood samples, it was 15.8% (21/133). Out of *C. burnetii*-positive ticks, there were double infections by *Borrelia* species and *C. burnetii* in *H. dromedarii* and *Am. lipidium* and triple infections at one *Am. cohaerens* tick (*C. burnetii*,* Borrelia* spp., and *Babesia microti*). In addition, two positive camel blood samples were found to carry *C. burnetii* with *Borrelia* spp. Our research findings indicate the presence of *Coxiella burnetii* among camels and their associated ticks in Egypt and emphasize the potential of having coinfection. To prevent the transmission of this infection to other animal species or humans, appropriate control measures should be implemented.

## Introduction

Query fever (Q fever or Coxiellosis) is one of the most common neglected zoonotic diseases caused by the *Coxiella burnetii* (*C. burnetii*) bacterium (Devaux et al. 2020). *C. burnetii*, an intracellular gram-negative bacterium, occurs worldwide except in New Zealand and Antarctica (Eldin et al. 2017; Klemmer et al. 2018; Nimo-Paintsil et al. 2022). Domestic ruminants, birds, and ticks are reservoirs for the disease and serve as a source of infection for people (De Bruin et al. 2013; Selim et al. 2018; Rerkyusuke et al. 2024). *C. burnetii can transmit* transstadially and transovarially through most soft and hard ticks. (Mediannikov et al. 2010). Over forty ticks and at least fourteen species of soft ticks have been identified as carriers of *C. burnetii*. This indicates the potential role of these arthropods in the spread of the bacterium across regions such as Argentina, Egypt, and Ethiopia. (Eldin et al. 2017; Loftis et al. 2006; Pacheco et al. 2013). *C. burnetii* pathogenicity increases after tick-borne transmission (Cutler et al. 2007). It multiplies in tick guts or stomachs, resulting in large loads of live organisms ejected with feces, saliva, and coxal fluid, which contributes significantly to the proliferation of *C. burnetii* in the environment (Mediannikov et al. 2010; Norlander 2000). In animals, Q fever is usually asymptomatic. However, it can cause miscarriage in the late stages of pregnancy and decrease reproductive efficiency due to stillbirth and delivery of weak infants (Li et al. 2018). Human infection occurs mainly by inhalation of contaminated air. The clinical features of Q fever involve flu-like symptoms but, in severe cases, cause pneumonia and granulomatous hepatitis (Duron et al. 2015). In Egypt, as in many other underdeveloped nations, Q fever is not a notifiable disease, even though seroprevalences of *C. burnetii* can reach up to 32% in adults, 22% in children, and 16% among farmers and vets (Klemmer et al. 2018). *Coxiella burnetii* serosurvey examinations in herds and farms in Africa, North Africa, the Arabian Peninsula, and Asia revealed that dromedary camels had much higher seroprevalence than other ruminants (Devaux et al. 2020). A study of 2,699 animals in Egypt found that only camels had a high seropositivity rate of 40.7% (215/528) for *C. burnetii* and that bacterial DNA was also detected in various ticks collected from them (Abdelbaset et al. 2023). However, insufficient data regarding the molecular survey of *C. burnetii* in Egypt, and the role of camels and associated ticks in transmitting this bacterium to humans and animals remains unclear. Consequently, the primary aim of this study is the molecular detection of *C. burnetii* in camels and associated ticks in Egyptian slaughterhouses.

Another significant issue in the epidemiology of tick-borne diseases (TBDs) is coinfection (multispecies infections) (Pawełczyk et al. 2021). Ticks can obtain various pathogenic species (such as parasites, bacteria, or viruses) either by directly feeding on different vertebrate hosts or by co-feeding with infected ticks on the same host, which acts as a “bridge” and allows uninfected ticks to acquire the infection (Voordouw 2015). Coinfection in humans and animals may increase illness severity and substantially impact the treatment and diagnosis of TBDs (Diuk-Wasser et al. 2016; Pawełczyk et al. 2021). Recent studies indicate that Egyptian camels and their ticks may be infected with *Borrelia* species and the zoonotic *Babesia microti* (Ashour et al. 2023a, b). Coinfections of *Babesia microti* and *Borrelia burgdorferi* are commonly found in Ixodes ticks, which are known to transmit these pathogens to humans. This coinfection can result in more severe or long-lasting symptoms in humans (Belongia 2002; Hersh et al. 2014). Co-infection in ticks is an important area of research, as it can help inform public health interventions and improve our understanding of the epidemiology of TBDs. So, this study also identifies the potential coinfections of *C. burnetii* with *Borrelia* spp. and *Babesia microti.*

## Methods

### Study area and sample collection

**Fig. 1:**
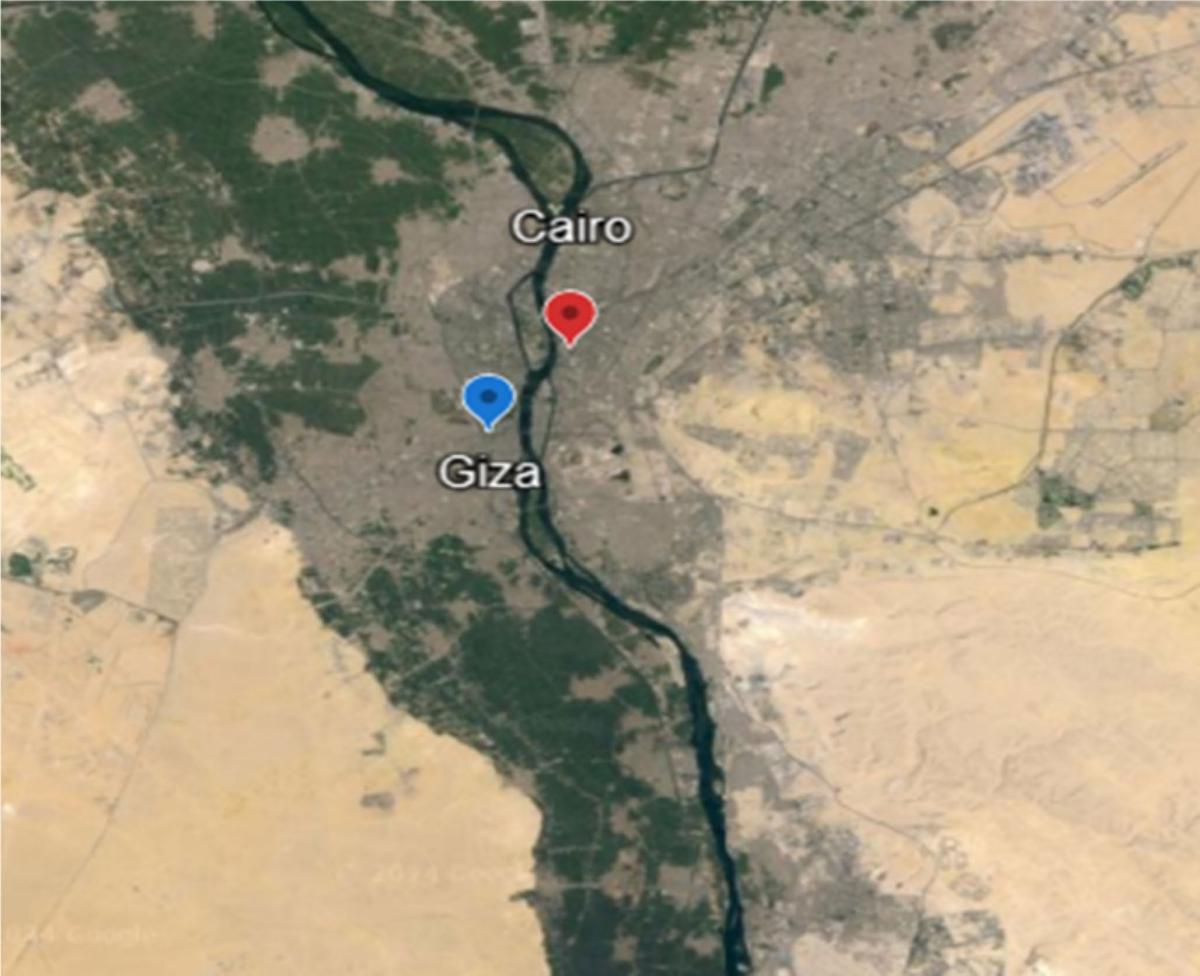
Tick collection sites from the central Egyptian slaughterhouses (Cairo and Giza governments). These governorates are located at 30°02’52"N to 31°14’05"E, 29°59′13″N to 31°12′42″E. Source: Google Earth V. 2.0, Jan 2004

Tick infestation was determined in one-humped camels (*Camelus dromedaries*) aged > 3 years. Samples of adult hard ticks and blood were taken from infested camels from the central Egyptian slaughterhouses (Cairo and Giza governments). These governorates are located at 30°02’52"N to 31°14’05"E, 29°59′13″N to 31°12′42″E. Source: Google Earth (version 2.0, Jan 2004) (Fig. 1). A total of 133 blood samples were collected from the jugular vein of camels in sterile vacuum tubes and mixed with ethylenediaminetetraacetic acid (EDTA) buffer, and 1260 adult hard ticks were removed by blunt end forceps, gripped the base of the capitulum to avoid losing mouthparts of ticks. Each camel’s ticks were stored in individual, prelabeled capped bottles on ice, awaiting identification.

### Tick identification

Ticks were counted and then identified to the genus level using a direct stereomicroscope. It is important to identify every tick specimen based on reliable keys and compare it with the voucher reference specimens according to (Estrada-Peña et al. 2004, 2013). Then, collected ticks were differentiated molecularly by amplifying the *COX1* gene and subsequent sequencing according to (Abdullah et al. 2016; Ashour et al. 2023b).

### DNA extraction

A Thermo Scientific GeneJET Genomic DNA Purification Kit (Thermo Fisher, Darmstadt, Germany) was used to extract DNA from whole camel blood samples and hard ticks. After following the manufacturer’s directions, the extracted DNA was stored at − 20 °C.

### Molecular detection of *C. burnetii*

Camel blood and tick DNA were screened for the presence of *C. burnetii* using nested PCRs based on the *htpAB* -IS1111   gene (Boden et al. 2012). The primers (Metabion, Germany) used and the thermocycling conditions are listed in Table 1. Each PCR included both positive and negative controls. PCR carried out on a Techne^®^ prime Thermal Cycler, UK. The PCR products were electrophoresed on a 1.5% agarose gel and seen under UV light. PCR products of representative samples were purified using the Qiaquick purification Kit (Qiagen, Germany) according to the manufacturer’s instructions, and sequenced with the forward primer *IS111*F1. The sequencing was conducted using the Big Dye Terminator V3.1 Sequencing Kit (Applied Biosystems). The sequences obtained were submitted to GenBank and compared to those accessible in the NCBI database using BLAST analysis.

**Table 1:** Primers and thermocycling conditions for identifying *C. burnetii*

Target gene	Primer sequences (5′-3)	Thermal cycles	Bp	Reference
*htpAB* **- IS1111**	IS111 F1: TACTGGGTGTTGATATTGC IS111 R1: CCGTTTCATCCGCGGTG	95 °C, 3 min; 40 cycles (95 °C, 30 s; 52 °C, 30 s; 72 °C, 1 min), 72◦C, 4 min	485	**(**Boden et al. 2012)
	IS111 F2: GTAAAGTGATCTACACGA IS 111 R2: TTAACAGCGCTTGAACGT	95 °C, 3 min; 30 cycles (95 °C 30 s; 52 °C, 30 s; 72 °C, 30 s), 72 °C, 4 min	260	

### Detection of the *C. burnetii* coinfection by nested PCR

An additional nested PCR was run targeting IGS (16–23 sRNA) for *Borrelia* spp. and *ß-tubulin* genes for *Babesia microti* on positive *C. burnetii* blood and tick samples to detect the coinfection. Primers and PCR conditions described in previous studies were used according to (Ashour et al. 2023a, b; Li et al. 2020; Reiter et al. 2015).

### Phylogenetic analysis

The BLAST analysis of GenBank was used to assess the degree of similarity with previously published sequences. (http://blast.ncbi.nlm.nih.gov/). The CLUSTALW 1.8^®^ program was used to calculate multiple sequence alignments and similarities after modifying the length of the sequence with the BioEdit sequence alignment editor (v. 7.0.9.0). The phylogenetic trees were constructed using the neighbor-joining approach with 1000 bootstrap repetitions in MEGA X software (Kumar et al. 2018). The evolutionary distances were computed using the Maximum Composite Likelihood model.

### Statistical analysis

Prevalence data were described in tables as percentages and 95% confidence intervals for proportions. Data were analyzed using Fisher’s Exact test (*FET*) to test the relationship between the prevalence of *C. burnetii* and the tick species. Significance was set at *P* ≤ 0.05. The statistical analysis was performed using PASW Statistics for Windows (Released 2009, Version 18.0., Chicago: SPSS Inc.) software.

## Results

### Ticks

All collected ticks belonged to the Ixodidae family (hard ticks) and were morphologically classified into 2 genera, *Hyalomma* (H) and *Amblyomma* (Am). Subsequently, the tick spp. infested camels were confirmed by amplifying and sequencing the *cox1* gene. The identified tick species included *H. dromedarii*,* H. marginatum*,* H. anatolicum*,* Am. lipidium*,* Am. cohaerens.*

### Prevalence of *C. burnetii*

The prevalence of *C. burnetii* in blood samples was 15.8% (21/133). But among adult ticks, it was 1.3% (16/1260) (Table 2). *C. burnetii* was detected in *H. dromedarii* (12/880, 1.4%), *H. marginatum* (1/297, 0.3), *H. anatolicum* (1/66, 1.5%), *Am. lepidum* (1/11, 9.1%) and *Am. cohaerens* (1/6, 16.7%) (Table 3), and the relationship between the prevalence of *C. burnetii* among tick species was significant (*FET* = 12.62, *P* = 0.013).

**Table 2:** Detection of *C. burnetii* by nested PCR in tick and camel blood samples and total coinfection rates

Type of samples	Total examined samples	No. of positive *C. burnetii* (%)	*C. burnetii *coinfected with		Total
			*Borrelia *spp. (%)	*Borrelia *spp. + *Babesia microti *(%)	
**Blood**	133	21 (15.8)	2 (9.5)	-	2
**Tick**	1260	16 (1.3)	2 (12.5)	1 (6.3)	3

**Table 3:** Prevalence of *C. burnetii* and coinfections of *C. burnetii with Borrelia* spp. and *Babesia microti* in the examined samples

Samples		Examined samples	*C. burnetii* positive samples No. (%)	95% Confidence Interval (95% CI)	Coinfection	
					Double infection (*C. burnetii* + *Borrelia *spp.)	Triple infection (*C. burnetii* +* Borrelia *spp. & *Babesia microti*)
**Camels**	Blood	133	21 (15.8)	10.49–23.01%	2	-
**Tick spp.**	*H. dromedarii*	880	12 (1.4)	0.75–2.40%	1	-
	*H. marginatum*	297	1 (0.3)	< 0.01–2.08%	-	-
	*H. anatolicum*	66	1 (1.5)	< 0.01–8.88%	-	-
	*Am. lipidium*	11	1 (9.1)	< 0.01–39.91%	1	-
	*Am. cohaerens*	6	1 (16.7)	1.14–58.22%	-	1
**Total examined ticks**		**1260**	**16 (1.3)**	**0.77–2.07%**	**2**	**1**

### Coinfections in camel blood and tick samples

Among 16 ticks positive for *C. burnetii*, there were occurrences of double infections involving *Borrelia* species and *C. burnetii in H. dromedarii* and *Am. lipidium* ticks, accounting for 12.5% (2/16). Additionally, a triple infection was observed in one *Am. cohaerens* tick, involving *C. burnetii*, *Borrelia* spp., and *Babesia microti*, constituting 6.3% (1/16). Furthermore, two camel blood samples were found to carry *C. burnetii* along with *Borrelia* spp., representing 9.5% (2/21) of the samples analyzed (Table 3).

### Sequencing and phylogenetic analysis

*Coxiella burnetii* infection was verified through the partial sequencing of the IS1111 gene from five representative positive tick samples and two positive camel blood samples. The gene sequences were submitted to GenBank, with the accession numbers OL790415, OK391174, OL790414, OL840474, OL845918, OK391175, and OL790416.

**Fig. 2:**
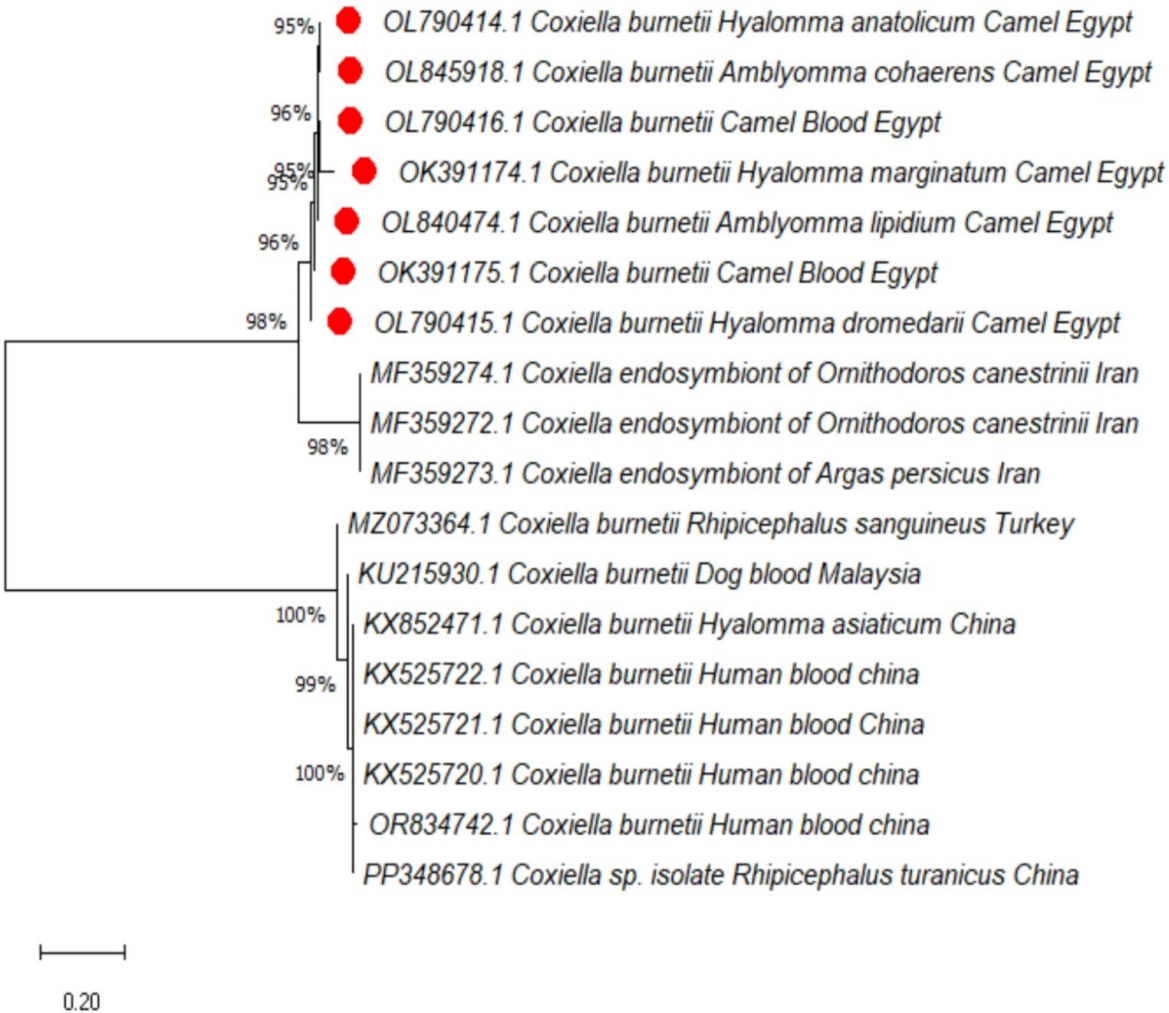
Phylogenetic tree based on the htpAB-IS1111 gene sequences of *C. burnetii.* The trees were constructed and analyzed by the neighbor-joining method. A small red dot indicates the new sequences provided by the present study

BLAST analysis revealed that All Egyptian strains isolated in this study were grouped with the second cluster of strains isolated from humans in China (Fig. 2).

## Discussion

Coxiellosis is a disease with significant veterinary field and public health implications. It mostly results in major financial losses for animal producers as well as the country’s economy (Kamaly et al. 2022). The presence of Q fever in camels has been recorded in Chad, Tunisia, Sudan, Saudi Arabia, the UAE, Egypt, and Ethiopia (Selim and Ali 2020). However, the molecular detection of Q fever in Egypt is scarce, especially in camels and associated ticks. Our analysis found *C. burnetii* DNA in 15.8% of the camel blood samples. In previous studies, the prevalence rates of *C. burnetii* in Egypt, Iran, Saudi Arabia, and Kenya ranged from 13 to 46%, 15.9%, 10.76%, and 18.6, respectively (Selmi et al. 2019). These infected camels may play a role in the epidemiology of Q fever. As a result, it is necessary to refocus attention on Q fever because of its severe effect on human health and agricultural systems. *Coxiella burnetii* DNA was found in 12 *H. dromedarii*, which are the predominant ixodid ticks found on camels in Egypt, and this tick species may help spread *C. burnetii* among dromedary herds (Abdullah et al. 2016; Gharbi et al. 2013). An Egyptian study using PCR on the *IS30A* spacer found that the prevalence of *C. burnetii* was 5.3% in *H. dromedarii* (Abdullah et al. 2018). Ticks in Tunisia were found to have *C. burnetii* DNA in 5.7% of *H. impeltatum* and 1.9% of *H. dromedarii*. This means that the *C. burnetii* found in *H. dromedarii* ticks that live on camels is probably zoonotic and harmful in these areas (Selmi et al. 2019). In Germany, the pathogen was detected in 19 of 1000 *Ixodes ricinus* ticks (1.9%) based on the transposase element *IS1111* and the isocitrate dehydrogenase gene (Hildebrandt et al. 2011b). In our study, *C. burnetii* was identified in several tick species; these findings agree with prior research suggesting that tick infestation increases the risk of Q fever infection (Benaissa et al. 2017; Toledo et al. 2009). The phylogenetic analysis, using the *IS1111* gene, revealed that all obtained sequences were similar to the pathogenic *C. burnetii* strains isolated from humans in China. This finding suggests that *C. burnetii* strains found in camel and infested ticks are probably zoonotic. Therefore, additional research is necessary to confirm the hypothesis that ticks infected with *C. burnetii* can transmit the disease to domestic animals and humans. Ticks that feed on camels can carry multiple pathogens, increasing the possibility of coinfections, complicating clinical presentations, and misinterpretation of various diseases affecting camels (Barghash et al. 2016; Chen et al. 2014). In a study conducted in Germany by (Hildebrandt et al. 2011a), they detected coinfections with *Coxiella burnetii*. Specifically, one nymph exhibited a triple coinfection with *C. burnetii*, *Borrelia burgdorferi* s.l., and *Borrelia garinii*. Additionally, among 1,000 *Coxiella*-positive ticks, some were also found to be infected with *Borrelia* spp., spotted fever group rickettsiae, or *Babesia* spp., alongside their *C. burnetii* infections (Teodorowicz and Weiner 2022). In China, there have only been a few reports that *Borrelia burgdorferi* s.l. coinfected with other diseases in ticks where Lyme disease is endemic (Chen et al. 2014). Further research on *Babesia microti* and *Borrelia burgdorferi* infections in rats and ticks has shown that coinfection with these pathogens is prevalent in vectors and enzootic hosts, with a higher probability of coinfection than predicted by chance (Diuk-Wasser et al. 2016; Pawełczyk et al. 2021). According to recent research, camels and associated ticks can harbor *Borrelia* and *Babesia microti* as a single infection (Ashour et al. 2023a, b). The new finding in the present study is that there were double infections by *Borrelia* species and *C. burnetii* in *H. dromedarii* and *Am. lipidium* ticks, while one *Am. cohaerens* ticks exhibited triple coinfection with *C. burnetii*,* Borrelia* spp., and *Babesia microti*. Coinfection by these pathogens may increase disease severity if transmitted by these ticks, which increasingly threatens human and animal health. In addition, two camel blood samples were found to carry *C. burnetii* with *Borrelia* spp. The co-infection of individuals with both *Babesia* and *Borrelia* species can significantly impact the clinical course, particularly in non-immunocompetent patients. Diagnosis can be challenging because both diseases manifest with nonspecific symptoms like fever, fatigue, and flu-like illness (Moniuszko et al. 2014). So, the coinfection in ticks and their hosts needs further investigation into the dynamics and risks associated with these interactions, due to their potential impact on human and animal healthcare.

## Conclusion

Based on the results of molecular detection of *C. burnetii* in Egyptian camels’ blood and ticks, this is alarming and suggests that camels infested with ticks might be playing a crucial role in the epidemiology of the disease. Additionally, some ticks were found to carry multiple pathogens; specifically, two were infected with both *Borrelia* species and *C. burnetii* as in *H. dromedarii* and *Am. lipidium* species, while another was simultaneously infected with triple pathogens, *C. burnetii*, *Borrelia* spp., and *Babesia microti*. Therefore, both doctors and vets must be aware of potential coinfections when dealing with tick bites to prevent misdiagnoses and increased severity of diseases.

## Data Availability

All the data generated in this study are included in this published article. The sequences of the *C. burnetii* IS1111 gene were deposited in GenBank, [Accession No. OL790415, OK391174 OL790414, OL840474, OL845918, OK391175, and OL790416].
